# Clinician Perspectives on Telemedicine: Observational Cross-sectional Study

**DOI:** 10.2196/29690

**Published:** 2021-07-09

**Authors:** Maria Alcocer Alkureishi, Zi-Yi Choo, Gena Lenti, Jason Castaneda, Mengqi Zhu, Kenneth Nunes, George Weyer, Julie Oyler, Sachin Shah, Wei Wei Lee

**Affiliations:** 1 Department of Academic Medicine University of Chicago Chicago, IL United States; 2 Pritzker School of Medicine Chicago, IL United States; 3 Department of Medicine University of Chicago Chicago, IL United States; 4 Department of Obstetrics and Gynecology University of Chicago Chicago, IL United States; 5 Department of Pediatrics University of Chicago Chicago, IL United States

**Keywords:** telemedicine, clinician perspective, patient-centered care, burnout, trainee, outpatient, workflow, virtual health, training, human factors

## Abstract

**Background:**

Since the COVID-19 pandemic onset, telemedicine has increased exponentially across numerous outpatient departments and specialties. Qualitative studies examining clinician telemedicine perspectives during the pandemic identified challenges with physical examination, workflow concerns, burnout, and reduced personal connection with patients. However, these studies only included a relatively small number of physicians or were limited to a single specialty, and few assessed perspectives on integrating trainees into workflows, an important area to address to support the clinical learning environment. As telemedicine use continues, it is necessary to understand a range of clinician perspectives.

**Objective:**

This study aims to survey pediatric and adult medicine clinicians at the University of Chicago Medical Center to understand their telemedicine benefits and barriers, workflow impacts, and training and support needs.

**Methods:**

In July 2020, we conducted an observational cross-sectional study of University of Chicago Medical Center faculty and advanced practice providers in the Department of Medicine (DOM) and Department of Pediatrics (DOP).

**Results:**

The overall response rate was 39% (200/517; DOM: 135/325, 42%; DOP: 65/192, 34%); most respondents were physicians (DOM: 100/135, 74%; DOP: 51/65, 79%). One-third took longer to prepare for (65/200, 33%) and conduct (62/200, 32%) video visits compared to in-person visits. Male clinicians reported conducting a higher percentage of telemedicine visits by video than their female counterparts (*P=*.02), with no differences in the number of half-days per week providing direct outpatient care or supervising trainees. Further, clinicians who conducted a higher percentage of their telemedicine by video were less likely to feel overwhelmed (*P=*.02), with no difference in reported burnout. Female clinicians were “more overwhelmed” with video visits compared to males (41/130, 32% vs 12/64, 19%; *P=*.05). Clinicians 50 years or older were “less overwhelmed” than those younger than 50 years (30/85, 35% vs 23/113, 20%; *P=*.02). Those who received more video visit training modalities (eg, a document and webinar on technical issues) were less likely to feel overwhelmed by the conversion to video visits (*P=*.007) or burnt out (*P=*.009). In addition, those reporting a higher ability to technically navigate a video visit were also less likely to feel overwhelmed by video visits (*P=*.02) or burnt out (*P=*.001). The top telemedicine barriers were patient-related: lack of technology access, lack of skill, and reluctance. Training needs to be focused on integrating learners into workflows. Open-ended responses highlighted a need for increased support staff. Overall, more than half “enjoyed conducting video visits” (119/200, 60%) and wanted to continue using video visits in the future (150/200, 75%).

**Conclusions:**

Despite positive telemedicine experiences, more support to facilitate video visits for patients and clinicians is needed. Further, clinicians need additional training on trainee education and integration into workflows. Further work is needed to better understand why gender and age differences exist. In conclusion, interventions to address clinician and patient barriers, and enhance clinician training are needed to support telemedicine’s durability.

## Introduction

Telemedicine uses electronic communications and software, like video and telephone visits, to deliver remote clinical services to patients [1]. Positive telemedicine outcomes include increased access to care, reduced wait times, improved clinical outcomes, and high patient and clinician satisfaction [2,3]. Despite this, telemedicine is generally less accepted by clinicians compared to patients [4,5], citing concerns over compensation, inadequate training, additional work, and difficulty adapting to technology [5].

Since the COVID-19 pandemic onset, telemedicine has increased exponentially across numerous outpatient departments and specialties [6,7]. Clinicians had to quickly pivot to provide substantial amounts of virtual care, resulting in the need to learn new workflows. In qualitative studies examining clinician telemedicine perspectives in the pandemic’s wake, clinicians reported challenges with physical examination, workflow concerns, burnout, and reduced personal connection with patients [8,9]. Although these studies set a baseline for understanding clinician barriers to telemedicine, they only explored perceptions of a relatively small number of physicians [8,10] or were limited to physicians from one specialty [9,11-13]. Furthermore, few studies assessed clinician perspectives on effectively integrating trainees into telemedicine workflows, an important area to address to support the clinical learning environment. As telemedicine maintains its foothold in outpatient medicine throughout and likely beyond the pandemic, it is necessary to understand a broad range of clinician perspectives on its impact on patient care, workflows, and trainee education, particularly since clinicians are more satisfied with telemedicine when they have input and support in its development [3].

Our study aims to survey pediatric and adult medicine clinicians at the University of Chicago Medical Center (UCMC) to understand perspectives on telemedicine benefits and barriers, workflow impacts, and training and support needs. Capturing clinician perceptions in various outpatient departments and specialties is critical to improving the clinician and patient telemedicine experience and to ensuring successful integration and durability of virtual encounters [14].

## Methods

### Setting and Participants

UCMC is a large urban academic health system and affiliated care network that provides tertiary care in the South Side of Chicago. As background, telemedicine was used for outpatient primary care at UCMC in the Department of Medicine (DOM) and Department of Pediatrics (DOP) whenever possible beginning March 15, 2020, to provide safe and socially distanced care. The total number of UCMC ambulatory visits at this time dropped substantially, with ambulatory visits falling to 23% of visit volumes when compared to the same week in the fiscal year (FY) 2019 [7]. After approximately 6 weeks, however, UCMC ambulatory visit volume had reached 92% of FY 2019 volumes, largely driven by the increase in virtual visits by nearly 1000 of our ambulatory clinicians. Overall, between March 15 and May 31, 2020, UCMC virtual visits increased from 0 to 48,475 visits; 60.5% of total ambulatory visits were virtual, of which 61.2% (n=29,661) were by video and 38.8% (n=18,814) were by telephone [7].

### Survey Development

We developed a 54-question survey (Multimedia Appendix 1) to capture clinician perceptions and needs for telemedicine implementation. Questions were based on a literature review of the impact of telemedicine on patient and clinician satisfaction and workflows, and informed by discussions with key UCMC stakeholders and leaders, practicing clinicians, and trainees. The survey consisted of Likert-style and open-ended questions, and assessed key areas including perceptions about benefits and barriers (n=20), workflow impacts (n=5), overall satisfaction (n=4), and training or support needs (n=6). Clinicians who worked with trainees (eg, medical students, residents, or fellows) were asked about their experiences with trainee integration and education (n=7). Open-ended questions (n=4) were included to elicit suggestions not previously asked. This project received a formal Determination of Quality Improvement project status according to UCMC institutional policy and, as such, was not reviewed by an institutional review board.

### Survey Distribution 

In July 2020, 517 UCMC physicians and advanced practice providers (APPs; eg, advanced practice nurses, clinical nurse specialists, and physicians’ assistants) in the DOM (n=325) and DOP (n=192) were invited via email to participate in the survey. The email was sent by UCMC leadership and the study investigators (MAA and WWL). Data was collected and managed using REDcap (v8.9.2; Vanderbilt University) [15]. The survey was open for 6 weeks, with one reminder email at 3 weeks. Individual emails were sent to DOP and DOM chairs and section chiefs at regular intervals, notifying them of their response rate and asking them to encourage clinician participation.

### Data Analysis

REDcap data was exported to Stata 16 (Stata Corp) [16] and RStudio (version 3.6.1; RStudio, PBC) [17] for statistical analysis. Quantitative outcomes were summarized by descriptive statistics. Chi-square tests, Fisher exact tests, and *t* tests assessed differences in outcomes among groups of interest. Ordinal logistic regressions examined associations between ordinal outcomes and explanatory variables of interest. Significance was defined as a two-sided *P* value less than .05.

Open-ended question responses were collectively pooled and read. Content analysis identified unique response themes, and representative quotations were identified to build a picture of clinicians’ collective experiences and video visit needs [18].

## Results

### Overview

The overall response rate was 39% (200/517; DOM: 135/325, 42%; DOP: 65/192, 34%). Respondent demographics are displayed in Table 1. The majority of respondents were faculty physicians (DOM: 100/135, 74%; DOP: 51/65, 78.5%), with roughly a quarter of APP respondents (DOM: 35/135, 26%; DOP: 14/65, 21.5%; *P*<.001). Most clinicians were aged 30 to 59 years (154/200, 77%), and 65% (130/200) were female. More female clinicians were also younger (83/130, 64% females <50 years vs 27/64, 42% males; *P=*.006). There were no significant differences by department (DOM vs DOP) or clinician age in terms of the number of half-days per week spent providing direct outpatient care, supervising trainees, or the percentage of telemedicine visits they personally conducted by video in the past week (Table 2). Although there were gender differences, with more male clinicians reporting they conducted a higher percentage of telemedicine visits by video than their female counterparts (*P=*.02), there were no significant differences in number of half-days per week spent providing direct outpatient care or supervising trainees.

**Table 1:** Clinician information by department.

Variables			Department of Medicine (n=135), n (%)		Department of Pediatrics (n=65), n (%)		*P* value	
**Clinician position**								.77
	Physician	100 (74.1)		51 (78.5)				
	Advanced practice provider^a^	35 (25.9)		14 (21.5)				
**Age (years)**								.65
	20-29	2 (1.5)		0 (0.0)				
	30-39	48 (35.6)		17 (26.2)				
	40-49	28 (20.7)		18 (27.7)				
	50-59	27 (20.0)		16 (24.6)				
	60-69	23 (17.0)		11 (16.9)				
	≥70	6 (4.5)		2 (3.1)				
**Gender**								.49
	Female	85 (63.0)		45 (69.2)				
	Male	47 (34.8)		17 (26.2)				
	Prefer not to say	3 (2.2)		3 (4.6)				
**Half-days per week providing direct outpatient care^b^**								.07
	0-2	56 (41.5)		16 (24.6)				
	3-4	44 (32.6)		22 (33.8)				
	5-6	24 (17.8)		15 (23.1)				
	≥7	11 (8.1)		11 (16.9)				
**Telemedicine visits personally conducted by video in the past week?^b^ (%)**								.09
	0-24	38 (28.1)		26 (40.0)				
	25-49	33 (24.4)		9 (13.8)				
	50-74	32 (23.7)		10 (15.4)				
	≥75	32 (23.7)		20 (30.8)				
**Number of half-days per week spent supervising trainees^c^**								.21
	0	69 (51.1)		34 (52.3)				
	1-2	51 (37.8)		19 (29.2)				
	≥3	14 (10.4)		12 (18.5)				
**Types of video visit training received**								
	Received a document on technical issues	83 (61.5)		47 (72.3)		.18		
	Webinar on technical issues	47 (34.8)		17 (26.2)		.29		
	In-person training on technical issues	5 (3.7)		3 (4.6)		.72		
	Received a document on communication strategies	35 (25.9)		24 (36.9)		.15		
	Webinar on communication strategies	18 (13.3)		9 (13.8)		>.99		
	In-person training on communication strategies	1 (0.7)		2 (3.1)		.25		
	None	25 (18.5)		6 (9.2)		.14		
	Other	4 (3.0)		3 (4.6)		.68		

^a^Examples of advanced practice providers include advanced practice nurses, clinical nurse specialists, and physicians’ assistants.

^b^Refers only to visits conducted personally by the clinician and not trainees they supervised.

^c^Trainees include medical students, residents, and fellows.

**Table 2:** Clinician information by age and gender.

Variables			Female (n=130), n (%)		Male (n=64), n (%)		*P* value			Younger than 50 years (n=113), n (%)			Older than 50 years (n=85), n (%)			*P* value	
**Half-days per week providing direct outpatient care^a^**								.13									.35
	0-2	39 (30.0)		30 (46.9)					41 (36.3)			31 (36.5)					
	3-4	44 (33.8)		21 (32.8)					38 (33.6)			28 (32.9)					
	5-6	33 (25.4)		5 (7.8)					24 (21.2)			14 (16.5)					
	≥7	14 (10.8)		8 (12.5)					10 (8.8)			12 (14.1)					
**Telemedicine visits personally conducted by video in the past week?^a^ (%)**								.02									.16
	0-24	47 (36.2)		15 (23.4)					39 (34.5)			25 (29.4)					
	25-49	30 (23.1)		12 (18.8)					24 (21.2)			17 (20.0)					
	50-74	28 (21.5)		11 (17.2)					27 (23.9)			14 (16.5)					
	≥75	25 (19.2)		26 (40.6)					23 (20.4)			29 (34.1)					
**Number of half-days per week spent supervising trainees^b^**								.52									.45
	0	69 (53.1)		29 (45.3)					63 (55.8)			39 (45.9)					
	1-2	43 (33.1)		27 (42.2)					35 (31.0)			35 (41.2)					
	≥3	18 (13.8)		8 (12.5)					15 (13.3)			11 (12.9)					
**Presence of burnout^c^**								.29									.01
	Yes	56 (43.1)		22 (34.4)					55 (48.7)			26 (30.6)					
	No	74 (56.9)		42 (65.6)					58 (51.3)			59 (69.4)					
**Converting in-person visits to video visits has resulted in feeling...**								.05									.02
	More overwhelmed	41 (31.5)		12 (18.8)					31 (27.4)			24 (28.2)					
	Similarly overwhelmed	59 (45.4)		28 (43.8)					59 (52.2)			31 (36.5)					
	Less overwhelmed	29 (22.3)		24 (37.5)					23 (20.4)			30 (35.3)					

^a^Refers only to visits conducted personally by the clinician and not trainees they supervised.

^b^Trainees include medical students, residents, and fellows.

^c^As defined by respondents own definition of *burnout*.

### Training

Most clinicians received some video visit training on technical issues (DOM: 93/135, 69%; DOP: 51/65, 78%), and fewer received telemedicine communication practice training (DOM: 42/135, 31%; DOP: 27/65, 42%; *P*<.001). There were no differences in training across gender, age, or departments.

### Comparison of Video Visits With In-person and Telephone Visits

**Figure 1:**
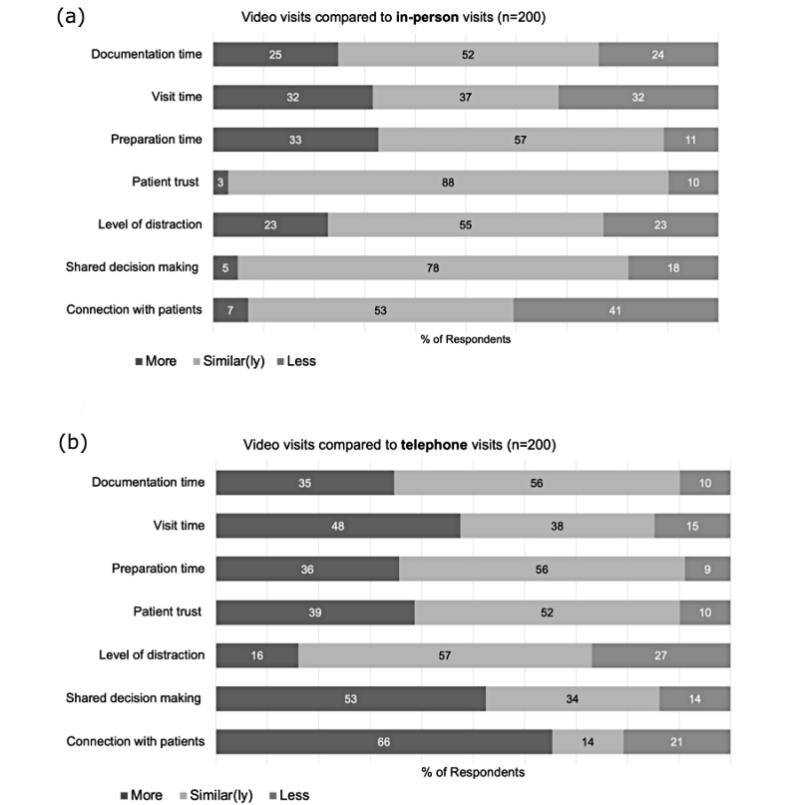
Video visit sentiments compared to in-person and telephone visits. Clinicians were asked to rate statements comparing video visits to (a) in-person visits and (b) telephone visits as “more,” “similar(ly),” or “less” in various categories (eg, “Video visits take more, similar, or less time to document compared to in-person visits”).

Figure 1 demonstrates clinician attitudes and experiences with regard to video, telephone, and in-person visits. Although nearly half of the 200 clinicians reported video visits took a similar amount of time to prepare (n=114, 57%) and document (n=104, 52%) compared to in-person visits, nearly one-third reported video visits took longer to prepare (n=65, 33%), conduct (n=64, 32%), and document (n=49, 25%). Likewise, when comparing video visits with telephone visits, nearly half reported video visits took a similar amount of time to prepare (n=111, 56%) and document (n=111, 56%). However, one-third of clinicians reported video visits took more time to prepare (n=72, 36%), conduct (n=96, 48%), and document (n=69, 35%) than telephone visits. Although there were no differences across gender or age, DOP clinicians were significantly more likely to report that video visits took longer to document compared to in-person visits (DOP: 25/65, 38% vs DOM: 32/135, 24%; *P=*.03) and telephone visits (DOP: 29/65, 45% vs DOM: 40/135, 30%; *P=*.04).

Despite the virtual nature of the visit, most of the 200 clinicians (n=156, 78%) felt they could promote shared decision making during video visits as well as they could in in-person visits. Half (n=106, 53%) felt they could better promote shared decision making during video visits compared to telephone visits. Just over half (n=105, 53%) felt they could personally connect as well or better with patients during video visits compared to in-person visits, with 66% (n=131) reporting they connected better with patients over video than over telephone. Although there were no differences across gender or age, DOP clinicians were more likely to report personal connection (DOM: 79/135, 59%; DOP: 52/65, 80%; *P=*.003), and the ability to share decisions with patients (DOM: 60/135, 44%; DOP: 46/65, 71%; *P=*.01) was better over video compared to telephone. DOP clinicians were also more likely to report that their ability to share decisions with patients was as good or better via video compared to in-person visits (DOM: 105/135, 78%; DOP: 60/65, 92%; *P=*.01).

Just over half of the 200 clinicians reported their level of distraction was similar when comparing video visits to in-person (n=110, 55%) and telephone visits (n=114, 57%). Most (n=176, 88%) felt patient trust in their diagnosis over video was similar compared to in person, whereas about half (n=104, 52%) felt patient trust over video was similar compared to telephone. Just over one-third (n=78, 39%) felt patient trust was better over video compared to telephone. Finally, nearly two-thirds agreed that being able to visualize a patient’s home environment (n=120, 60%) and being able to have patient companions join the video visit (n=168, 84%) added valuable insight into their patients’ lives. There were no differences across gender, age, or departments in these areas.

### Video Visit Barriers

The top three most commonly cited barriers from the 200 clinicians to conducting video visits were not clinician-specific barriers but rather patient related, including patient lack of technical knowledge (n=139, 70%), lack of patient access to necessary technology for a video visit (n=132, 66%), and patient reluctance to have a video visit (n=75, 38%; Table 3). The next most frequently cited barriers were inadequate staff support both during (n=70, 35%) and when scheduling visits (n=68, 34%). There were no differences across gender, age, or departments in visit barriers.

**Table 3:** Barriers and training needs.

Barriers		DOM^a^, n (%)	DOP^b^, n (%)
**Barriers to conducting video visits (DOM: n=135; DOP: n=65)**			
	Patient lack of technical knowledge	105 (77.8)	34 (52.3)
	Patient access to necessary technology	101 (74.8)	31 (47.7)
	Patient reluctance	61 (45.2)	14 (21.5)
	Inadequate staff support during visits	48 (35.6)	22 (33.8)
	Inadequate scheduling staff support	44 (32.6)	24 (36.9)
**Barriers to conducting video visits with residents/fellows (DOM: n=65; DOP: n=31)**			
	Concerns about integrating them into video visit workflows	34 (52.3)	18 (58.1)
	Uncertainty about documentation rules	12 (18.5)	10 (32.3)
	Other	14 (21.5)	7 (22.6)
**Barriers to conducting video visits with medical students (DOM: n=65; DOP: n=31)**			
	Concerns about integrating them into video visit workflows	26 (40.0)	10 (32.3)
	Uncertainty about documentation rules	15 (23.1)	4 (12.9)
	Unsure how to give performance feedback	9 (13.8)	3 (9.7)
	Patient reluctance to having medical students involved	9 (13.8)	2 (6.5)
	Other	7 (10.8)	5 (16.1)
**Training needs (DOM: n=135; DOP: n=65)**			
	Performing a video visit exam	62 (45.9)	35 (53.8)
	Billing aspects	51 (37.8)	31 (47.7)
	Technical aspects	52 (38.5)	22 (33.8)
	Communication strategies	33 (24.4)	22 (33.8)
	Integrating residents/fellows into visit workflows	38 (28.1)	26 (40.0)

^a^DOM: Department of Medicine.

^b^DOP: Department of Pediatrics.

Faculty physicians who precepted trainees (n=96) during telehealth visits cited “concerns about integrating them into video visit workflows” and “uncertainty about documentation rules” as the top two barriers both when working with residents and fellows (n=52, 54% and n=22, 23%, respectively) and medical students (n=36, 38% and n=19, 20%, respectively; Table 3). The next most commonly cited barriers for medical educator clinicians was uncertainty about how to give trainees feedback on their virtual visit performance (residents and fellows: n=15, 16%; medical students: n=12, 13%). Overall, nearly three-quarters of teaching clinicians agreed or strongly agreed that “virtual medicine has made clinical teaching more difficult” (n=69, 72%). This sentiment was further reflected in clinicians’ open-ended responses where some (n=8) reported having little experience with and needing substantially more training to integrate medical students, residents, and fellows into virtual workflows (Textbox 1). There were no differences across gender, age, or departments in trainee barriers.

**Themes and representative quotations of video visit needs.**

**How can your section/department best support you in the use of video visits?**

- Provide clinic staff support to prepare patients for visits
  - “I would like support staff dedicated to virtual visits, so they can interface with patients with expertise.”
  - “Technical support, and working with patients so they are comfortable with video visits.”
- Streamline scheduling processes and video visit workflows
  - “Video visit slots are like clinic slots; allow for enough time for the visit and documentation of the visit.”
  - “Screen the patients who benefit from the video visits, and who should have personal visits at clinic.”

**How can your section/department best support your patients in the use of video visits?**

- Provide technical support for patients
  - “Provide the support staff to help patients troubleshoot technical issues”
  - “Help them figure out how to access the links and help them troubleshoot so that they are ready to go at the time of their virtual appointment.”
- Provide technology access for patients
  - “Ensure they have access to adequate technology. Some patients don't even have enough cellphone minutes.”
  - “Make them accessible via phone. Most of my patients do not have laptops/tablets and need to use their phone”

**What suggestions do you have on how to successfully integrate trainee teaching into telehealth visit workflows?**

- Establish learner workflows
  - “We have the trainees begin the call as they would in clinic...then call the attending and ‘present’ the patient and then both join on the call to finish the visit.”
  - “I think it would be good if the trainee and attending could somehow go into a breakout room to discuss the assessment and plan without the patient.”
- Provide teaching training for preceptors
  - “Guide preceptors on how to do this best.”
- Provide more time within telehealth teaching schedules
  - “Give preceptors more time in the schedules to account for the additional time it takes to precept a student.”

**Please share additional comments, suggestions, or experiences regarding your video visit experience**

- Video visit experiences have been positive, and are useful for many clinicians and patients.
  - “When patients are comfortable with the technology, video visits work very well. In addition, for the most part, patient show rates are significantly higher. I would like to have the opportunity to continue to use telehealth in the future for certain patient visits.”
  - “My patients really like the video visits, however for some frail/elderly patients, it's been both a blessing and a curse.”
- Video visit limitations and utility for certain types of appointments
  - “The inability to perform at least a halfway good physical exam will eventually severely impact patient outcomes and increase cost to the system through increased testing.”
  - “I would support continuing video visits for 1. patients who live far away and are challenged by the distance, 2. patients who have limited resources to come to clinic 3. stable patients who don't need a detailed hands-on examination 4. discussion of a serious condition, or serious decision-making.”

### Clinician Experience, Burnout, and Satisfaction

In the survey, participants were asked to self-report their perceived level of burnout. Overall, 81 clinicians reported burnout, with significant differences between departments (DOP: 36/65, 56%; DOM: 45/135, 34%; *P=*.004) but not by gender (male: 22/64, 34%; female: 56/130, 43%; *P=*.29). Of note, clinicians younger than 50 years (55/113, 49%) also reported higher levels of burnout compared to those 50 years or older (26/85, 31%; *P=*.01).

Participants were also asked whether converting in-person visits to video made them feel less, similarly, or more overwhelmed. Overall, only 28% (n=56) of the 200 clinicians felt more overwhelmed, with nearly half of clinicians (n=90, 45%) feeling similarly and 27% (n=53) feeling less overwhelmed. Notably, a higher proportion of female clinicians (41/130, 32%) than males (12/64, 19%) reported feeling more overwhelmed (*P=*.05). Although there was no overall difference between clinician age and feeling *more* overwhelmed with video visits, clinicians 50 years or older felt significantly *less* overwhelmed (30/85, 35%) than those younger than 50 years (23/113, 20%; *P=*.02). Differences in feeling overwhelmed by video visits were not seen across departments.

With respect to training, clinicians who received a greater number of video visit training modalities (eg, a document and webinar on technical issues) were less likely to feel overwhelmed by the conversion to video visits (*P=*.007) or burnt out (*P=*.009). Those reporting a higher ability to technically navigate a video visit were also less likely to feel overwhelmed by video visits (*P=*.02) or burnt out (*P=*.001). Further, clinicians who conducted a higher percentage of their telemedicine by video were less likely to feel overwhelmed (*P=*.02); however, there was no difference in reported burnout. There were no gender, age, or departmental differences in training or self-reported ability. Interestingly, there were also no significant differences in feeling burnt out or overwhelmed by the switch to video visits and the number of either personal or supervising teaching attending clinic sessions per week or by the type of virtual visits their trainees had (eg, video or phone).

Overall, more than half of the 200 clinicians (n=119, 60%) enjoyed conducting video visits, and 69% (n=137) reported “the benefits of video visits outweighed the negatives.” Most wanted to continue using video visits (n=150, 75%), which was higher than the fraction of clinicians (n=85, 43%) who wanted to continue using telephone visits (*P*<.001). There were no differences across gender, age, or department in these areas.

### Support and Training Needs

In terms of clinician resources for technical or clinical support during video visits, the largest percentage of the 200 respondents said they had no resource to go to when an issue (technical or process) occurred (n=73, 37%), with the next largest group citing patient service representatives (n=50, 25%) or medical assistants (n=37, 19%) as their primary support resource. The top three video visit training needs reported were guidance on performing an exam (n=97, 49%), billing (n=82, 41%), and technical aspects (n=74, 37%; Table 3). There were no significant differences across gender, age, or department in training needs.

These sentiments were reflected in the open-ended responses (n=42) in Textbox 1. At the departmental level, clinicians (n=14) called for improved staff support before and during video visits. Regarding patient-facing barriers, they also described the need for patient technical support (n=13), while others (n=9) reiterated the need for improved patient technology literacy and access to ensure successful virtual visits. Finally, clinicians shared additional comments regarding their video visit experience. Despite overall positive experiences, clinicians (n=13) commented on video visit challenges such as adjusting to new virtual workflows and the limitations of video visits for certain patient populations and visit types.

## Discussion

As virtual visits continue to comprise an important and increasingly prevalent form of health care delivery, it is important to understand the clinician experience and how they perceive video and telephone visits compared to in-person visits. Most clinicians enjoyed conducting video visits and felt that the connection they had with patients was similar to in-person visits. However, it is important to note that one-third of clinicians reported video visits took longer to prepare, conduct, and document compared to in-person visits. Prior to the COVID-19 pandemic, most of our clinicians had never conducted virtual visits. The overnight conversion to telemedicine required rapid adjustments to a new technology and the creation of new workflows. Further, with in-person visits, a medical assistant often starts the visit for the clinician, documenting intake questions and administering screening tools such as depression assessments, gathering background information such as interim hospitalizations and emergency room visits, and verifying information such as their medications, preferred pharmacy, and allergies.

At the start of the transition to telemedicine, most medical assistants were not assisting clinicians with these visit duties, and the burden of that additional workload and documentation fell to clinicians. Having conducted our study, the need to provide clinician visit support in the virtual setting much like that of the in-person setting became clear. Many clinicians stated that they needed more help supporting virtual visits so that patients could be *roomed* just like in a regular visit, and the lack of external visit support may have led to increased clinician burden and therefore increased time to prepare and document virtual visits. Additionally, we found clinicians who had more video visit training and higher self-rated technical knowledge were less likely to feel overwhelmed or burnt out. The longer time needed to prepare and document virtual visits could be due to the need for more training and increased familiarity with technology. As clinicians become more comfortable with virtual visits and new clinical support is implemented, providers should be resurveyed on whether they feel that telehealth visits take more time and what, if any, training needs they continue to have.

Prior to the pandemic, electronic documentation demands on clinicians were already high with clinicians spending more than one half of their workday, nearly 6 hours, interacting with the electronic health record (EHR) during and after clinic hours, 1 to 2 hours of which was during their personal time each night [19], an activity one author aptly termed “pajama time” charting [20]. Even more worrisome is that EHR documentation burden is linked to increases in medical errors, threats to patient safety, inferior documentation quality, job attrition, and clinician burnout [21]. With telemedicine potentially adding to this out-of-visit documentation load and total visit time, it is critical for institutions to recognize that increased demands on clinician time may increase burnout and to proactively develop interventions to promote efficient telemedicine workflows and EHR efficiency to minimize clinician burden and prioritize wellness.

Despite one-third of clinicians reporting it took longer to prepare, conduct, and document telemedicine visits, we found no significant differences in burnout or feeling overwhelmed by the conversion from in-person to video and clinicians’ personal or teaching attending workload. This may be partly due to the fact that our survey period was relatively early on in the course of the pandemic, and although data showing burnout is increasing [22], this may be due to the sustained impacts of the pandemic, and because of our survey time period, these rates may have not yet started to rise to the level that they are at now. There may also be an impact of infection risk during COVID-19 and burnout as a result of clinician anxiety and stress related to either personally contracting COVID-19 or passing it to a family member [23]. The reduced number of in-person visits at the start of the pandemic, which coincided with our study period, could have led to lower rates of burnout since working from home decreased clinician exposure risk and may have reduced infection-related stress and anxiety, thereby outweighing the potential burden of virtual visits themselves.

Further, COVID-19 significantly increased the challenges of work-life balance for clinicians with children [24]. School-aged children transitioned to remote learning, and many day cares and after-school programs closed, creating a sudden need for clinician parents to source childcare. This was a major stressor for many clinician parents, and although nonideal, telemedicine provided a way for clinicians with children to work from home. The ability to provide childcare in light of the pandemic may have led to lower rates of observed burnout.

Additionally, the finding that clinicians who had more video visit training and, perhaps consequently, a higher self-rated technical facility with video visits were less likely to feel overwhelmed or burnt out by transitioning to video visits underscores the importance of clinician familiarity and efficiency with technology as a key driver in their experience. Studies examining EHR use support this finding and suggest that enhanced education and training can improve clinician technical proficiency, self-reported efficiency, and satisfaction, which could eventually have an effect on burnout [25].

We also found that clinicians who conducted a higher percentage of their telemedicine visits by video were less overwhelmed. Although we know this variable refers to the *proportion* of telemedicine visits conducted by video, it is possible that these individuals also conducted a higher *amount* of visits by video by the time they took our survey. Perhaps this group of clinicians had become more adept at conducting video visits and therefore felt less overwhelmed moving their clinics to virtual because of their skill, as previously mentioned. However, it also may be that video visits are for some reason less stressful to conduct compared to telephone visits, perhaps because communication and assessment is easier with the added visual benefit of video. That said, further study in this area is needed.

Additionally, we saw differences between groups in regard to the burden of telemedicine and potential for subsequent burnout. For instance, women reported being more overwhelmed with video visits compared to men. This may be attributed to females conducting a lower percentage of telemedicine visits by video, which was shown to be associated with feeling overwhelmed, as previously mentioned. It is unclear why female clinicians were less likely to conduct video visits despite similar clinical and teaching workloads; however, given female clinicians were younger and thus more junior, they may have opted for fewer video and more telephone sessions. Further, prior to COVID-19, female physicians spent significantly more time on household activities and childcare than their male counterparts, which was likely exacerbated by the closing of schools, day cares, etc during the pandemic [26-28]. There is also evidence that female physicians are more likely to be in frontline clinical positions, less represented in high-level decision making roles [26] and that, overall, female physicians suffered from reduced publishing productivity during COVID-19 compared to male physicians [29]. The cognitive load of new virtual workflows along with these other pandemic-related stressors [30] may provide an explanation for the differences we found in our study between males and females.

Our study also found that older clinicians (>50 years) reported being less overwhelmed than younger clinicians with the addition of video visits to their practice, despite having a similar personal and teaching clinic workload as their younger counterparts, and that younger clinicians had higher burnout at baseline compared to older clinicians. Of note, other studies have similarly found older clinicians generally experienced greater well-being and lower levels of stress compared to younger clinicians during the pandemic [31,32]. Although we had anticipated that older clinicians would potentially be more overwhelmed with the introduction of new technologies to their practice, it may be that, in addition to the diminished childcare responsibilities previously mentioned, older clinicians have greater experience and trust in their diagnostic skill and long-standing relationships with their patient panels, allowing them to more smoothly transition their practice to a virtual setting. Conversely, younger clinicians may have higher rates of burnout due to lack of experience [31] and the need to balance childcare needs in the setting of school and day care closures. These differences underscore the need for health care organizations to understand the various stressors uniquely affecting their clinicians during the pandemic and beyond, and to invest in telemedicine support structures to reduce additional burden placed on clinicians.

Although our surveyed clinicians found they could still promote patient-centered care through virtual visits, we found notable differences between pediatric and adult medicine clinicians in these areas. In particular, pediatric clinicians found telephone visits less beneficial for connecting with and making shared decisions with their patients. Pediatric patients are often not participating verbally in the visit themselves, but rather the child’s parent or guardian; therefore, the added benefit of visually observing and connecting with the child through the camera may be more important on the pediatric side. That said, pediatric clinicians reported higher confidence compared to adult clinicians that they could share decision making with their patients over video compared to in-person visits. This reinforces the idea that, although virtual visits are still useful for pediatric patients, video visits may allow for more patient-centered techniques compared to telephone visits.

Although clinicians recognized the need for ongoing training for themselves, the top three telemedicine barriers clinicians encountered were not clinician-centered barriers such as inadequate staff support. Instead, the top three barriers identified were their patient’s barriers: access to technology, technical literacy and knowledge, and overall reluctance toward video visits. These findings have several important implications for patient care for telemedicine to be a successful means of providing care for all patients, not only technically savvy or resource-rich patients. Our findings underscore the need to better understand and minimize potential disparities with respect to the digital divide or the gap between persons who have and do not have access to new forms of information technology [33]. An early evaluation of telemedicine visits at UCMC, where Black or African American patients completed significantly fewer video visits but more telephone visits compared to White patients [7], helps further highlight this need. At other institutions, older patients, Black and Hispanic patients, patients with Medicaid insurance, and patients who need an interpreter were also less likely to have a video visit [34,35].

This finding is particularly troubling, as telemedicine was a lifeline for many to access needed clinical care during the pandemic. Telemedicine exposed inequities related to the digital divide for many of our South Side Chicago patients, and in response to this study and the knowledge that our clinician experience and success with telemedicine was critically dependent on our patient’s ability to access and use technology, we developed patient-facing materials to help patients prepare for and navigate virtual visits, including high-tech (portal, website, videos, email) and lower-tech (text, phone calls, paper mailing) means. We have also started a qualitative study in response to understand our patients’ telemedicine experiences [36] and will use our findings to expand our outreach, identify and develop needed patient resources and interventions to enhance access to technology, and better screen for and promote eHealth literacy. To minimize the digital divide, it is critical for organizations to further explore their patients’ telehealth experiences and engage them in helping identify the barriers they face that limit their ability to successfully participate in video visits [37]. In our study, clinicians reported challenges with integrating trainees into telemedicine workflows. Many were uncertain about how to document telemedicine encounters with trainees and how to provide performance feedback. As trainees return to the outpatient setting, it is necessary to address these barriers and to help teaching clinicians define opportunities for trainee education. As new telehealth competencies from the Association of American Medical Colleges emerge [38], clinician educators should focus on how to practically integrate these lessons into learner curriculum and practice. Finally, clinicians self-identified the need for further training and guidance on performing exams on video visits and technical and billing aspects of video visits. Given that over one-third of clinicians did not have a top resource for technical or process issues that arose during video visits, it is important to promote ongoing awareness and support for our many technical resources. In response, we implemented a telemedicine curriculum for medical students, residents, and faculty focusing on helping patients navigate virtual visits while integrating patient-centered care principles and provided faculty with additional training on integrating trainees into virtual workflows in a meaningful and educational manner [39].

There are several important limitations of our study to note. First, our study is limited to one institution, situated in a largely underserved area. To increase generalizability, our survey was cross departmental, including representation from our affiliate care partners who practice in nonacademic and community-based settings. Additionally, it is possible that clinician responses were influenced by the specific telemedicine platform used at UCMC; other organizations may have different experiences based on other platforms. It is important to note, however, that our survey questions broadly targeted aspects of the clinician virtual visit experience without reference to the specific telemedicine platform used. Finally, we did not directly survey patients during this time and all identified barriers, challenges, and perceptions of telemedicine in this study are based solely on the clinician experience. This underscores the need to elicit these perceptions directly from patients to better understand their challenges and perceived benefits of telemedicine.

In conclusion, this is the first study to elicit perspectives on telemedicine from a wide range of faculty from the departments of medicine and pediatrics. Clinicians identified barriers to implementation, challenges to incorporating trainee education, and training needs that should be addressed to improve the telemedicine experience. Overall, it is encouraging that clinicians enjoy video visits and can connect with their patients similarly to in-person visits. However, it is concerning that for a third of clinicians, video visits took longer to prepare, conduct, and document. To support clinician wellness, institutions must more completely understand and support clinician needs. Regarding trainee education, training is needed to help clinician educators successfully integrate students and house staff into virtual workflows, assess learner telemedicine performance, and structure virtual clinic feedback. Most importantly, the top three barriers to successful telemedicine implementation identified by clinicians are patient barriers, highlighting the need to better understand patient perceptions toward video and telephone visits, and proactively address barriers that contribute to the digital divide. It is critical to address each of these needs to support the durability of telemedicine visits as a way to complement and augment the care patients receive in person and to ensure that both clinician and patient experiences are efficient, positive, and patient-centered.

## Appendix

Multimedia Appendix 1 Clinician Survey.

## Abbreviations

APP: advanced practice provider
DOM: Department of Medicine
DOP: Department of Pediatrics
EHR: electronic health record
FY: fiscal year
UCMC: University of Chicago Medical Center
