# Supplementary material for: Clinician Perspectives on Telemedicine: Observational Cross-sectional Study
Source: JMIR Hum Factors. 2021 Jul 9;8(3):e29690. doi: 10.2196/29690 (PMC8274680; doi:10.2196/29690)
Supplement: Multimedia Appendix 1 [file humanfactors_v8i3e29690_app1.pdf]

Clinician Telemedicine Survey

Thank you for completing the following survey.

It should take you about 10 minutes to complete, and will assist us in better understanding your telemedicine perceptions and needs.

Identifier. Please enter the first 3 letters of your mother's maiden name followed by the FOUR digits of the DATE you were born.

(Ex. If mother's maiden name is Jones and your birthdate is 04/24/1966, identifier would be jon0424).

____

Position:

- ◯ Attending
- ◯ Midlevel provider (i.e., APN, PA, etc.)
- ◯ Other

If other, please specify.

____

Where do you practice clinically?

- ◯ BSD faculty practice
- ◯ Care Network (e.g. Ingalls) or Affiliate practice

Department

- ◯ Department of Anesthesia and Critical Care
- ◯ Department of Family Medicine
- ◯ Department of Medicine
- ◯ Department of Neurology
- ◯ Department of Obstetrics and Gynecology
- ◯ Department of Ophthalmology and Visual Science
- ◯ Department of Orthopedic Surgery and Rehabilitation Medicine
- ◯ Department of Pathology
- ◯ Department of Pediatrics
- ◯ Department of Psychiatry and Behavioral Neuroscience
- ◯ Department of Radiation and Cellular Oncology
- ◯ Department of Radiology
- ◯ Department of Surgery
- ◯ Other

If other, please specify.

____

Section	◯ Cardiology<br>◯ Computational Biomedicine & Biomedical Data<br>◯ Dermatology<br>◯ Emergency Medicine<br>◯ Endocrinology, Diabetes & Metabolism<br>◯ Gastroenterology, Hepatology & Nutrition<br>◯ General Internal Medicine<br>◯ Genetic Medicine<br>◯ Geriatrics & Palliative Medicine<br>◯ Hematology/Oncology<br>◯ Hospital Medicine<br>◯ Infectious Diseases & Global Health<br>◯ Nephrology<br>◯ Pulmonary/Critical Care<br>◯ Rheumatology<br>◯ Other
Section	◯ General Obstetrics & Gynecology<br>◯ Maternal-Fetal Medicine<br>◯ Gynecologic Oncology (Female Cancer)<br>◯ Gynecology and Minimally Invasive Surgery<br>◯ Urogynecology and Reconstructive Pelvic Surgery<br>◯ Reproductive Endocrinology & Infertility<br>◯ Family Planning & Contraceptive Research<br>◯ Other
Section	◯ Orthopedic Surgery<br>◯ Physical Medicine and Rehabilitation<br>◯ Sports Medicine<br>◯ Spine Surgery<br>◯ Podiatry
Section	◯ Academic Pediatrics<br>◯ Immunology, Pulmonology & Sleep Medicine<br>◯ Cardiology<br>◯ Critical Care<br>◯ Dermatology<br>◯ Development & Behavioral Pediatrics<br>◯ Emergency Medicine<br>◯ Endocrinology<br>◯ Gastroenterology, Hepatology, and Nutrition<br>◯ Hematology/Oncology<br>◯ Hospital Medicine<br>◯ Infectious Disease<br>◯ Kennedy Center<br>◯ Neonatology<br>◯ Nephrology<br>◯ Neurology<br>◯ Rheumatology<br>◯ Other
Section	◯ Child and adolescent<br>◯ Adult<br>◯ Other

Section	◯ Abdominal Imaging<br>◯ Breast Imaging<br>◯ Musculoskeletal Radiology<br>◯ Neuroradiology<br>◯ Nuclear Medicine<br>◯ Pediatric Radiology<br>◯ Thoracic Imaging<br>◯ Vascular and Interventional Radiology<br>◯ Other
Section	◯ Cardiac Surgery<br>◯ Colon & Rectal Surgery<br>◯ General Surgery<br>◯ Neurosurgery<br>◯ Otolaryngology-Head & Neck Surgery<br>◯ Pediatric Surgery<br>◯ Plastic & Reconstructive Surgery<br>◯ Thoracic Surgery<br>◯ Transplant Surgery<br>◯ Trauma Surgery<br>◯ Urology<br>◯ Vascular Surgery<br>◯ Other
If CARE NETWORK, please indicate your section:	__________________________________
If BSD, please indicate your section if not already selected above:	__________________________________
Age (years)	◯ 20-29<br>◯ 30-39<br>◯ 40-49<br>◯ 50-59<br>◯ 60-69<br>◯ >70
Gender	◯ Female<br>◯ Male<br>◯ Non-binary<br>◯ Self-describe<br>◯ Prefer not to say
Self decribe:	__________________________________

On how many half-days per week do you provide direct outpatient care to patients (as opposed to precepting housestaff)?	◯ < 1 ◯ 1 ◯ 2 ◯ 3<br>◯ 4 ◯ 5 ◯ 6 ◯ 7<br>◯ 8 ◯ 9 ◯ 10 ◯ I don't have a clinic, but supervise trainees in clinic
In the past week, what percentage of your telemedicine visits have been conducted by video?	◯ 0-24% ◯ 25-49% ◯ 50-74%<br>◯ 75% or more
In the past week, what percentage of your telemedicine visits have been conducted by phone?	◯ 0-24% ◯ 25-49% ◯ 50-74%<br>◯ 75% or more
During video visits, how often do you currently ask your patient to perform specific physical exam maneuvers?	◯ Every visit<br>◯ Only if indicated<br>◯ Never
During video visits, how often do you currently use the "share screen" feature?	◯ Every visit<br>◯ Only if indicated<br>◯ Never
What have you used the "share screen" feature for? (Check all that apply)	☐ Verify medications, allergies, or problem list, etc.<br>☐ Show labs or imaging<br>☐ Document portions of the note with the patient (ie., HPI, Assessment and Plan)<br>☐ Show patient education resources (i.e., websites, videos,etc.)<br>☐ Summarize next steps<br>☐ Other
If other, please specify:	__________________________________
What Video Visit training have you received? (Check all that apply)<br><br>(Note: Technical training includes how to log on, start the visit, etc. Communication strategies training includes how to engage patients, non-verbal communication, etc.)	☐ Received a document on technical issues<br>☐ Webinar on technical issues<br>☐ In-person training on technical issues<br>☐ Received a document on communication strategies<br>☐ Webinar on communication strategies<br>☐ In-person training on communication strategies<br>☐ None<br>☐ Other
If other, please specify:	__________________________________

During a VIDEO VISIT, rate your ability to:

	Poor	Marginal	Acceptable	Good	Excellent
Technically navigate a visit.	○	○	○	○	○
Employ patient-centered communication strategies (i.e., maximizing non-verbal communication to promote connectedness and engagement).	○	○	○	○	○

What are YOUR barriers to conducting video visits? (Check all that apply)

- ☐ Lack of technical knowledge
- ☐ Lack of communications training
- ☐ Comfort level in performing video visit exams
- ☐ Difficulty accessing translation services
- ☐ Inadequate scheduling staff support
- ☐ Inadequate staff support during visits
- ☐ Patient lack of technical knowledge
- ☐ Patient privacy concerns
- ☐ Patient reluctance
- ☐ Patient access to necessary technology
- ☐ Other

If other, please specify: __________________________________

Compared to IN-PERSON visits....

	More	Similar(ly)	Less
I can connect _____ with my patients during video visits.	○	○	○
I can promote shared-decision making ______ during video visits.	○	○	○
I think my level of distraction is ________ during video visits.	○	○	○
I think my patients trust me ______ during video visits.	○	○	○
Video visits take me ______ time to prepare.	○	○	○
Video visits take me ______ time to conduct.	○	○	○
Video visits take me ______ time to document the visit.	○	○	○

Compared to TELEPHONE visits....

	More	Similar(ly)	Less
I can connect _______ with my patients during video visits.	◯	◯	◯
I can promote shared decision making _______ during video visits.	◯	◯	◯
I think my level of distraction is _______ during video visits.	◯	◯	◯
I think my patients trust me _____ during video visits.	◯	◯	◯
Video visits take me _____ time to prepare.	◯	◯	◯
Video visits take me _____ time to conduct.	◯	◯	◯
Video visits take me _____ time to document the visit.	◯	◯	◯

Rate your level of agreement with the following statements.

I can evaluate my patients more thoroughly via video than via phone visits.

- ◯ Strongly Agree
- ◯ Agree
- ◯ Neutral
- ◯ Disagree
- ◯ Strongly Disagree

Being able to visualize my patients' home surroundings on video visits allows me to gain important additional insight into my patients' lives.

- ◯ Strongly Agree
- ◯ Agree
- ◯ Neutral
- ◯ Disagree
- ◯ Strongly Disagree

Being able to have patient companions join the video visit allows me to gain important additional insight into my patients' lives.

- ◯ Strongly Agree
- ◯ Agree
- ◯ Neutral
- ◯ Disagree
- ◯ Strongly Disagree

Workflow

Which best describes the space in which you conduct video visits?

- ◯ Calm
- ◯ Somewhat calm
- ◯ Busy, but reasonable
- ◯ Somewhat chaotic
- ◯ Chaotic

Who is your primary resource when an issue (technical or process) occurs? (Check all that apply)

- ☐ Patient Service Representative
- ☐ Medical Assistant
- ☐ Nursing
- ☐ Practice Manager
- ☐ IT Service Desk
- ☐ None
- ☐ Other

If other, please specify.	__________________________________
Do you feel your technical or process issues are resolved quickly enough?	◯ Strongly Agree<br>◯ Agree<br>◯ Neutral<br>◯ Disagree<br>◯ Strongly Disagree
What is the ideal arrangement for your video visit schedule?	◯ Clinic sessions where I see ONLY video or ONLY in-person visits<br>◯ Clinic sessions where I see a MIX of in person and video visits<br>◯ Doesn't matter<br>◯ Other
If other, please specify:	__________________________________
Using your own definition of "burnout," please select ONE of the following:	◯ I enjoy my work. I have no symptoms of burnout.<br>◯ I am under stress and don't always have as much energy as I did, but I don't feel burned out.<br>◯ I am definitely burning out and have one or more symptoms of burnout, such as physical and emotional exhaustion.<br>◯ The symptoms of burnout that I'm experiencing won't go away. I think about frustrations at work a lot.<br>◯ I feel completely burned out and often wonder if I can go on.<br>◯ I am at the point where I may need to seek help.
Converting my in-person visits to video-visits has resulted in me feeling:	◯ More overwhelmed<br>◯ Similarly overwhelmed<br>◯ Less overwhelmed

Video Visits with Trainees

On how many half-days per week do you supervise trainees (medical students, residents, fellows) in clinic?	◯ < 1 ◯ 1 ◯ 2 ◯ 3<br>◯ 4 ◯ 5 ◯ 6 ◯ 7<br>◯ 8 ◯ 9 ◯ 10
In the past week, what percentage of your trainee's visits have been conducted by video?	◯ 0-24% ◯ 25-49% ◯ 50-74%<br>◯ 75% or more
In the past week, what percentage of your trainee's visits have been conducted by phone?	◯ 0-24% ◯ 25-49% ◯ 50-74%<br>◯ 75% or more

What are YOUR barriers to conducting video visits with medical students? (Check all that apply)

- ☐ N/A - I don't work with medical students
- ☐ Uncertainty about documentation rules
- ☐ Concerns about integrating them into video visit workflows
- ☐ Unsure how to give performance feedback
- ☐ Their lack of technical knowledge
- ☐ Their lack of communications training
- ☐ Patient reluctance to having medical students involved
- ☐ Other

If other, please specify: __________________________________

What are YOUR barriers to conducting video visits with residents/fellows? (Check all that apply)

- ☐ N/A - I don't work with residents/fellows
- ☐ Uncertainty about documentation rules
- ☐ Concerns about integrating them into video visit workflows
- ☐ Unsure how to give performance feedback
- ☐ Their lack of technical knowledge
- ☐ Their lack of communications training
- ☐ Their comfort level performing an exam
- ☐ Other

If other, please specify: __________________________________

If you DO NOT work with students or residents/fellows, PLEASE SELECT N/A for the following section.

When supervising VIDEO VISITS, rate your ability to:

	Poor	Marginal	Acceptable	Good	Excellent	N/A
Integrate medical students into visit workflows	○	○	○	○	○	○
Teach medical students visit best practices	○	○	○	○	○	○
Give medical students performance feedback	○	○	○	○	○	○
Integrate residents/fellows into visit workflows	○	○	○	○	○	○
Teach residents/fellows visit best practices	○	○	○	○	○	○
Give residents/fellows performance feedback	○	○	○	○	○	○

Virtual medicine has made clinical teaching more difficult.

- ○ Strongly Agree
- ○ Agree
- ○ Neutral
- ○ Disagree
- ○ Strongly Disagree

Overall Assessment and Feedback

I enjoy conducting video visits.

- ◯ Strongly Agree
- ◯ Agree
- ◯ Neutral
- ◯ Disagree
- ◯ Strongly Disagree

The benefits of video visits outweigh the negatives.

- ◯ Strongly Agree
- ◯ Agree
- ◯ Neutral
- ◯ Disagree
- ◯ Strongly Disagree

Post-COVID, I want to continue using video visits with patients .

- ◯ Strongly Agree
- ◯ Agree
- ◯ Neutral
- ◯ Disagree
- ◯ Strongly Disagree

Post-COVID, I want to continue using telephone visits with patients.

- ◯ Strongly Agree
- ◯ Agree
- ◯ Neutral
- ◯ Disagree
- ◯ Strongly Disagree

I would like more video visit training on (check all the apply):

- ☐ Technical aspects
- ☐ Billing aspects
- ☐ Communication strategies
- ☐ Performing a video visit exam
- ☐ Teaching best practices to medical students
- ☐ Integrating medical students into visit workflows
- ☐ Giving medical students performance feedback
- ☐ Teaching best practices to residents/fellows
- ☐ Integrating residents/fellows into visit workflows
- ☐ Giving residents/fellows performance feedback
- ☐ I do not need further training
- ☐ Other

If other, please specify:

____

How can your section/department best support YOU in the use of video visits?

____

How can your section/department best support YOUR PATIENTS in the use of video visits?

____

What suggestions do you have on how to successfully integrate trainee teaching into telehealth visit workflows?

____

Please share additional comments, suggestions, or experiences regarding your video visit experience:

____
